# Bronchoscopic Resection of High-Risk Tracheal Leiomyoma With Extracorporeal Membranous Oxygenation (ECMO) Standby: A Case Report Highlighting Multidisciplinary Approach

**DOI:** 10.7759/cureus.104331

**Published:** 2026-02-26

**Authors:** Jesse Liou, Ghassan Habib, Michael Wolf, Ronak Desai, Wissam Abouzgheib

**Affiliations:** 1 Pulmonary Medicine, Cooper University Hospital, Camden, USA; 2 Pulmonary Medicine, Cooper Medical School of Rowan University, Camden, USA; 3 Anesthesiology, Cooper University Hospital, Camden, USA; 4 Anesthesiology, Cooper Medical School of Rowan University, Camden, USA

**Keywords:** central airway obstruction, ecmo, interventional bronchoscopy, multidisciplinary care, tracheal leiomyoma

## Abstract

Tracheal leiomyomas are extremely rare benign tumors that are most commonly treated by bronchoscopic or surgical resection. Procedure-related complication rates are low, with surgical tracheal resection carrying the highest risk. Nonetheless, related airway obstruction is a potentially devastating complication. As proficiency develops, more centers are utilizing extracorporeal membranous oxygenation (ECMO) not just for critically ill patients but also for high-risk airway therapeutic procedures. However, ECMO carries its own potential complications, and the decision to cannulate requires multidisciplinary discussions. We present a case of a high-risk tracheal leiomyoma that was successfully resected bronchoscopically with ECMO on standby.

## Introduction

Tracheal tumors are rare, accounting for 0.1% of all tumors. These tumors are usually malignant, with a few exceptions. One such exception is a tracheal leiomyoma, a benign neoplasm that accounts for 1% of all tracheal tumors [1]. Tracheal leiomyoma is a rare, slow-growing, benign tumor, often causing a long delay in diagnosis because its symptoms, such as chronic cough, wheezing, and dyspnea, mimic common diseases like asthma or bronchitis. Due to the airway's large, compliant, and elastic nature, significant symptoms often do not manifest until the tumor has obstructed a large portion of the lumen, typically reported as more than 50% to 75% of the airway diameter [2]. Once discovered, diagnosis can be challenging due to the risk of airway occlusion during bronchoscopic or surgical management [3]. While there is no standard of care in managing central airway obstruction (CAO), the American College of Chest Physicians does recommend a comprehensive, multidisciplinary approach in preoperative planning [4]. The use of extracorporeal membranous oxygenation (ECMO) has increased exponentially over the past decade as a tool for management in operative airway procedures, tracheal obstruction, acute thoracic emergencies, and trauma [5]. Veno-venous extracorporeal membranous oxygenation (VV-ECMO) has been used to provide cardiopulmonary support in patients with difficult airways or in those at risk of complete airway obstruction. It functions by draining deoxygenated blood from a central vein, circulating it through a membrane oxygenator for gas exchange, and returning oxygenated blood to a separate central vein. In addition, VV-ECMO can emergently be deployed as a rescue maneuver for refractory hypoxemia [6]. This case report describes a high-risk tracheal mass, occluding >95% of the airway lumen, which was successfully removed without complication due to diligent multidisciplinary planning.

## Case presentation

**Figure 1:**
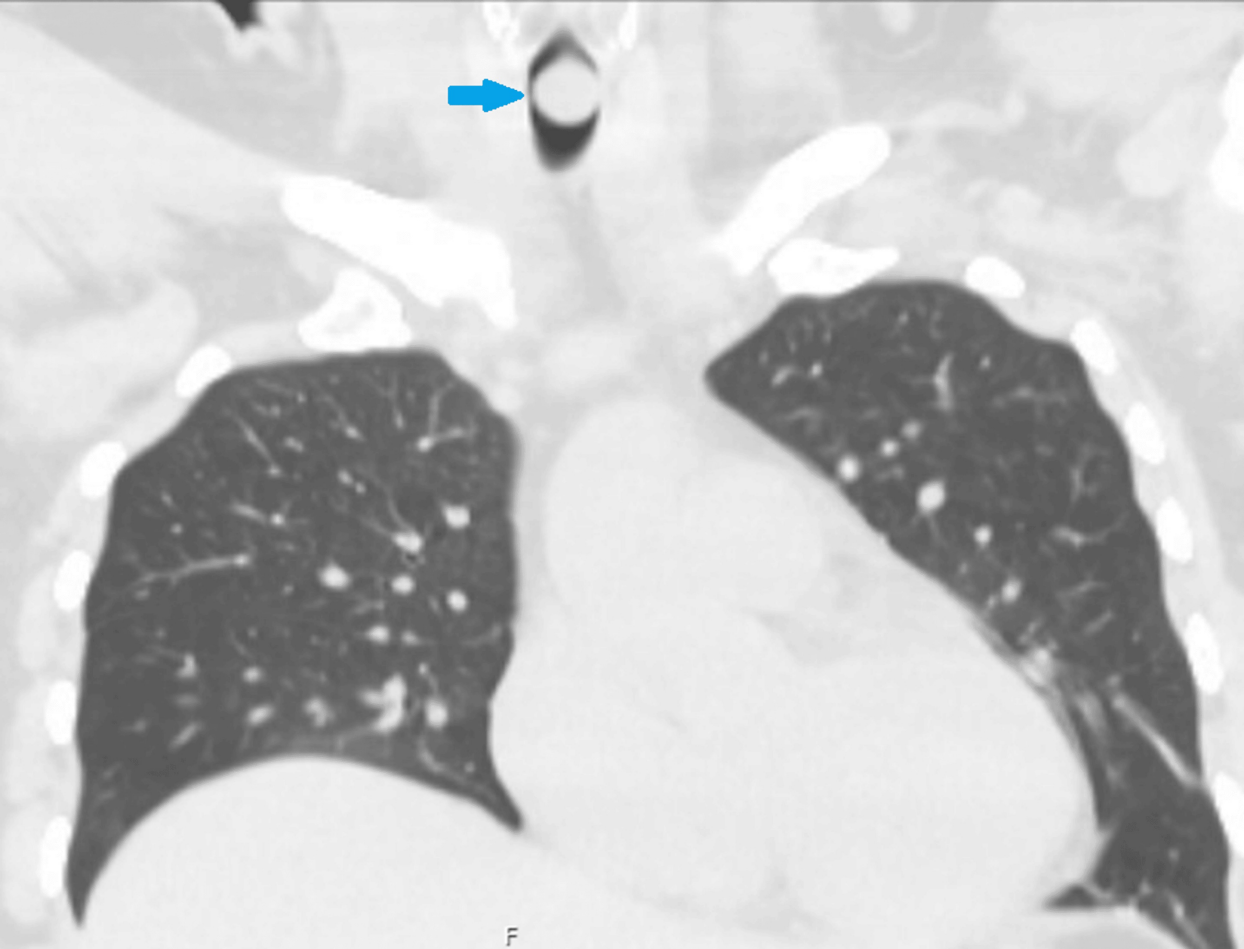
CT chest (lung window) of coronal cuts. Demonstrates severe tracheal narrowing secondary to a suspected tracheal mass (blue arrow).

**Figure 2:**
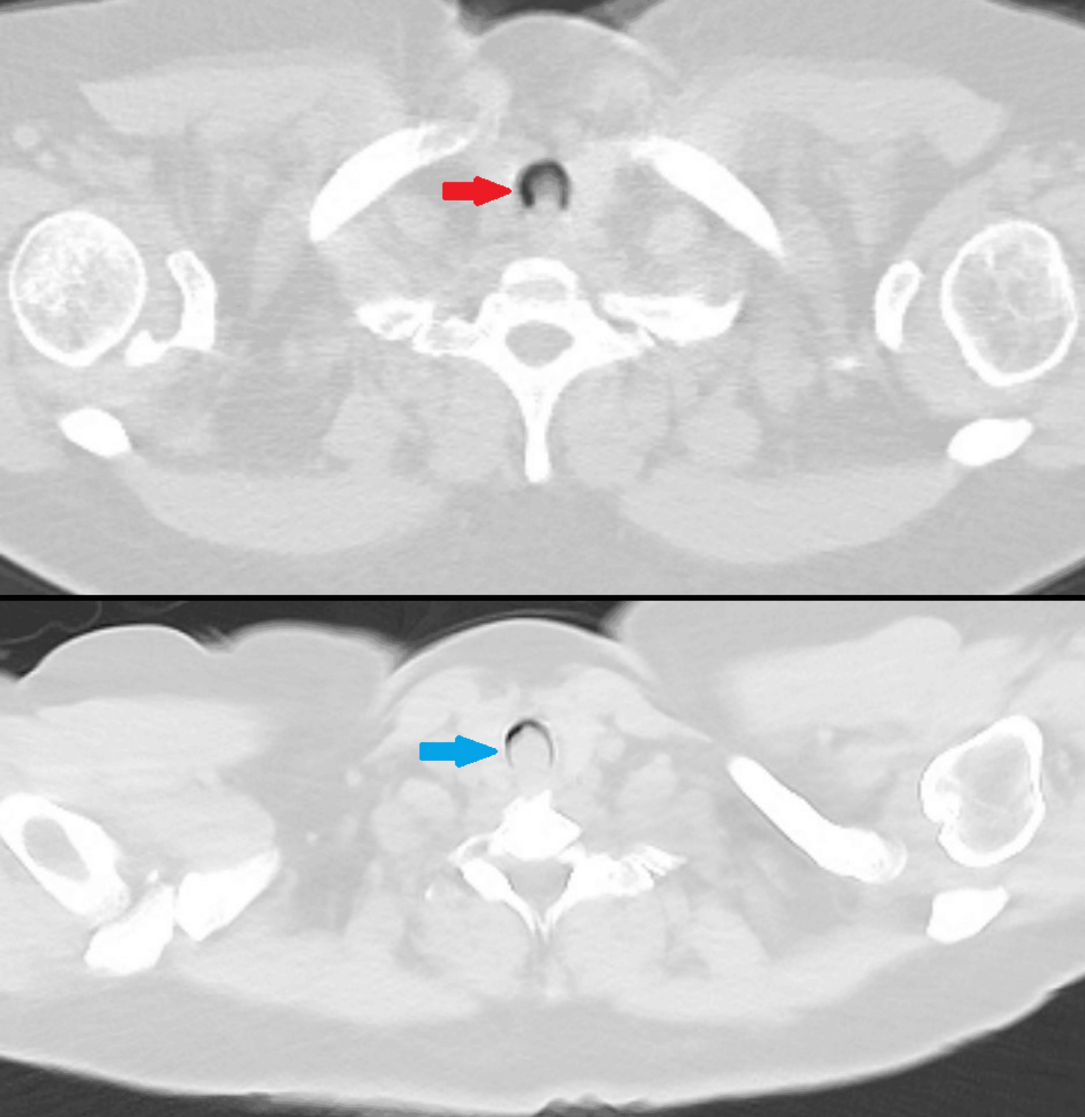
Computed tomography of the chest (lung window, axial cuts) of imaging 5 months prior. Top: depicting tracheal narrowing (red arrow) compared to imaging at the latest hospitalization; Bottom: depicting severe tracheal narrowing secondary to suspected tracheal mass (blue arrow).

A 51-year-old female with a notable medical history of asthma presented to her outpatient pulmonologist for a hospital follow-up, where tracheal narrowing was incidentally seen on a chest computed tomography (CT) scan (Figures 1, 2). She was hospitalized at the time for pain due to a fall, but did endorse ongoing wheezing and dyspnea that had persisted for three months following a prior COVID-19 infection. Chest x-rays were unremarkable for any tracheal or airway abnormalities. Her pulmonary function tests were all normal. She was maintained on maximal doses of triple inhaler therapy (budesonide/formoterol 160-4.5mcg twice per day, tiotropium bromide 2.5mcg daily, and albuterol 90mcg two to four times daily) and completed multiple rounds of corticosteroids and antibiotics. Review of prior CT imaging (Figure 2) demonstrated persistent severe tracheal narrowing, raising concerns for a tracheal mass.

She was referred to interventional pulmonary and was scheduled for bronchoscopy with possible hot snare and cryoprobe debulking. She was determined to be at high risk for decompensation due to the severity of tracheal narrowing and the possibility of complete occlusion of her airway. A multidisciplinary discussion between anesthesiology, cardiac surgery, pulmonology, and critical care determined that ECMO support would be available emergently if decompensation occurred.

**Figure 3:**
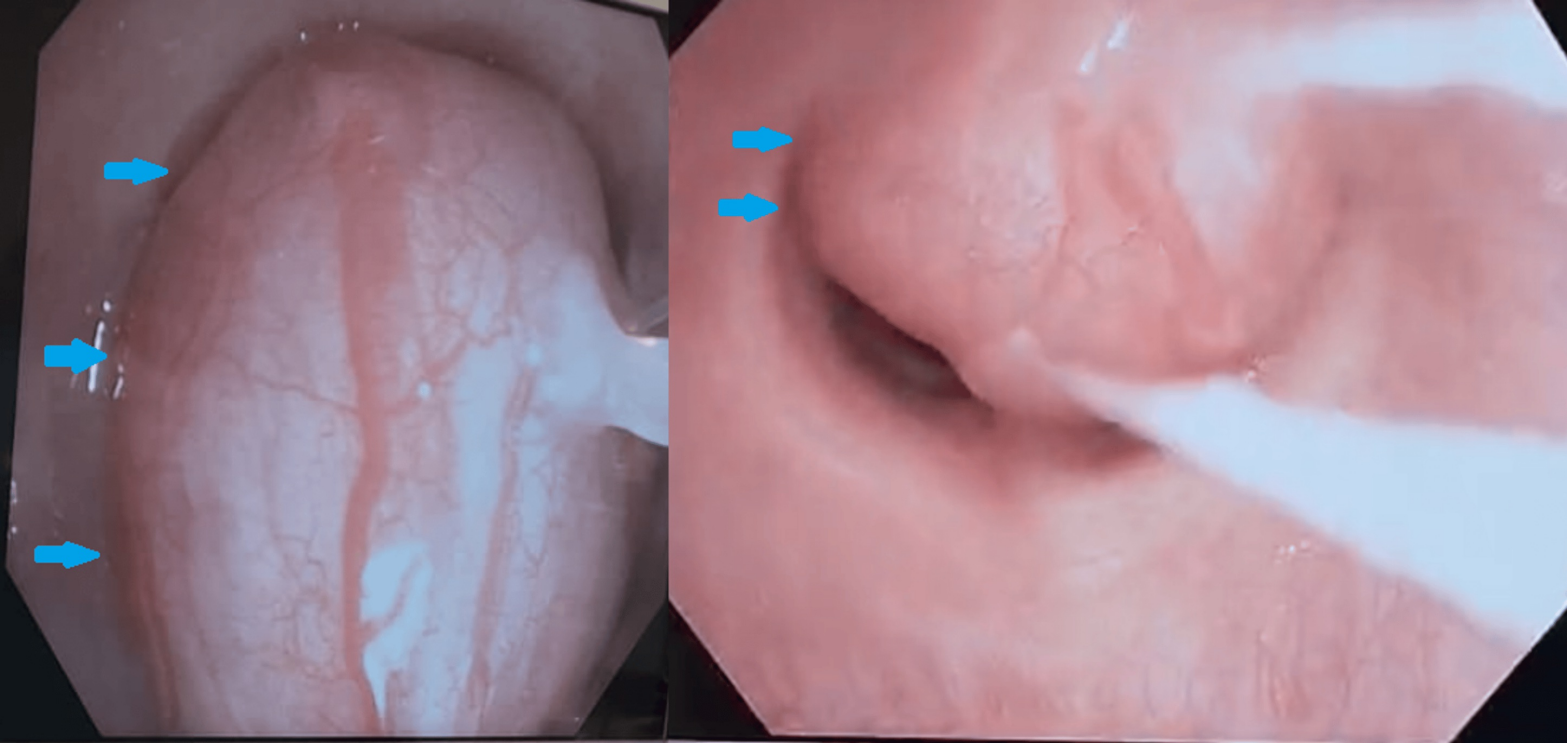
Mass (blue arrows) occluding 95% of tracheal lumen in two views: 0° (left) and rotated 90° (right).

**Figure 4:**
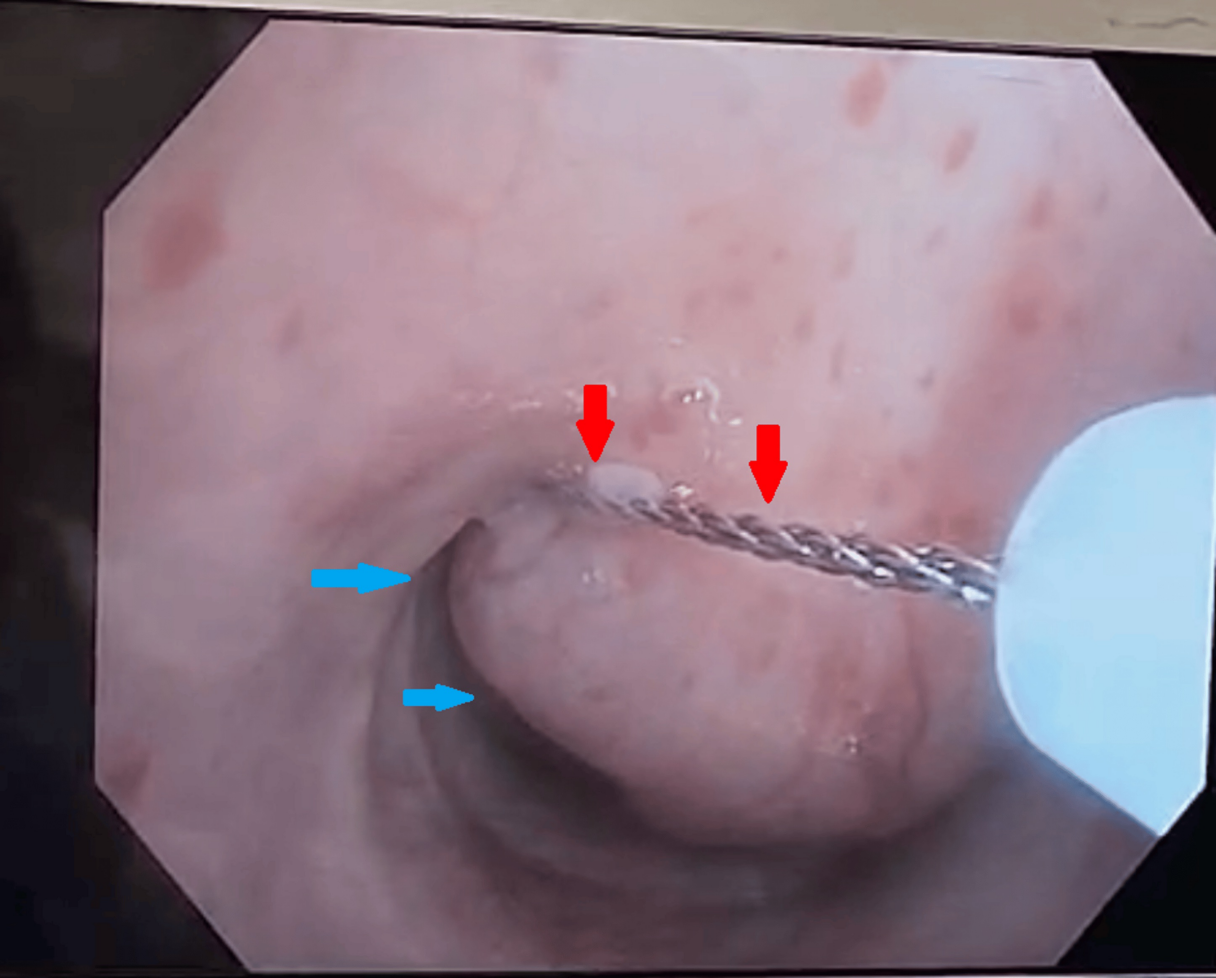
Hot snare (red arrows) wrapped around base of the lesion (blue arrows).

**Figure 5:**
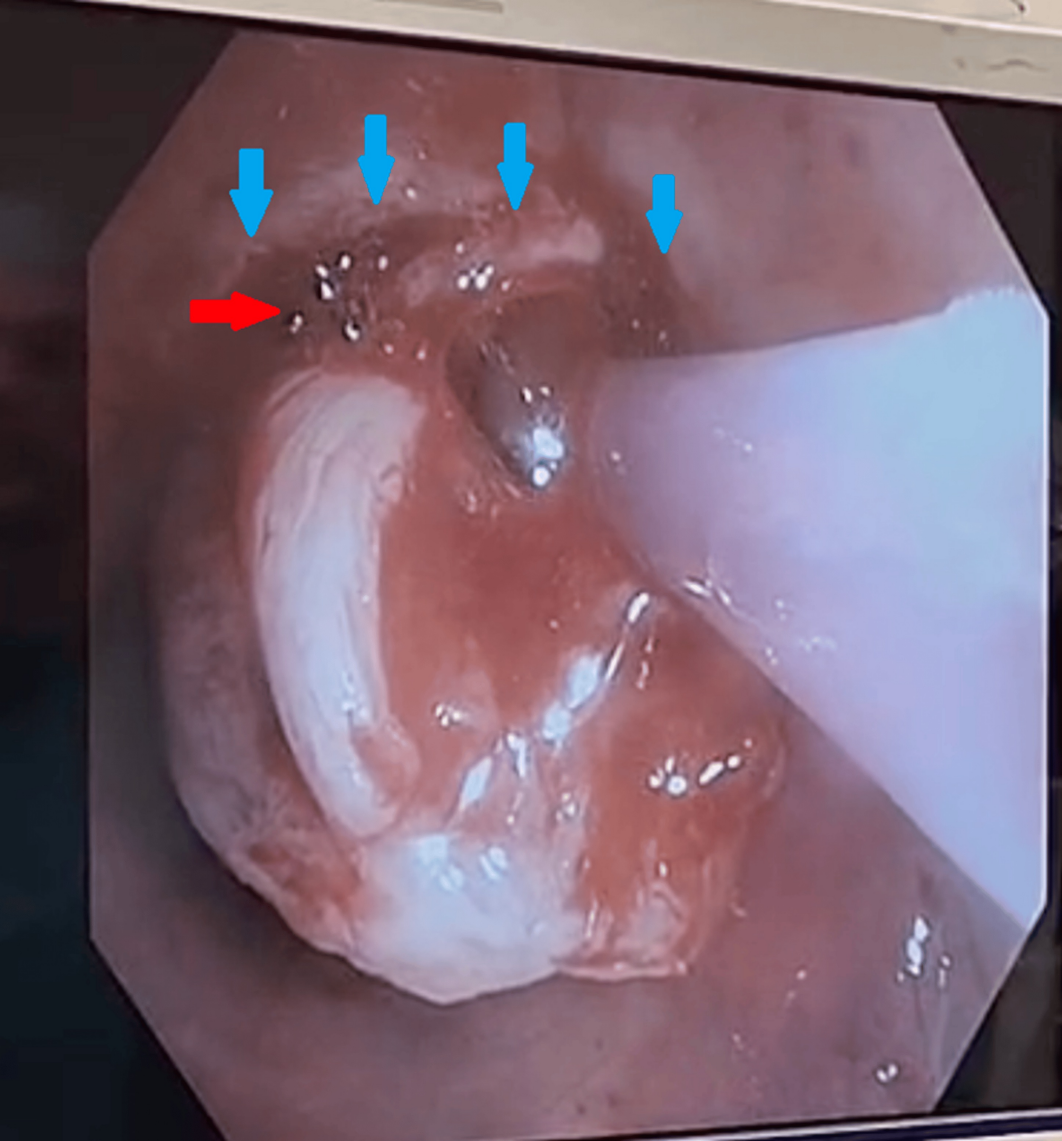
Hot snare (red arrows) wrapped around remainder of the tissue (blue arrows) at the base of stalk.

Preoperatively, the ECMO team marked the femoral vein sites, and the patient received both nebulized 2.25% racepinephrine 0.5mL and nebulized 0.083% albuterol 1.25mg. The anesthesiology team induced general anesthesia judiciously, utilizing a spontaneously ventilating technique with a mixture of propofol 180mcg/kg/min, remifentanyl 0.05mcg/kg/min, and dexmedetomidine 8mcg. Once adequately anesthetized, a therapeutic bronchoscope was used and inserted via a laryngeal mask airway (LMA). The trachea was entered, and a large, shiny, polypoid tracheal tumor was visualized, connected to the posterior wall and encompassing over 95% of the airway lumen (Figure 3). A hot snare was wrapped around the base of the lesion to slice through the stalk while coagulating associated vasculature (Figure 4). The freed lesion was deliberately displaced into the right main stem bronchus and was subsequently retrieved using the cryoprobe. There was no active bleeding upon inspection. The hot snare was then inserted again, and the remaining tissue at the base of the stalk was excised (Figure 5). Cryoprobe was utilized again for retrieval.

**Figure 6:**
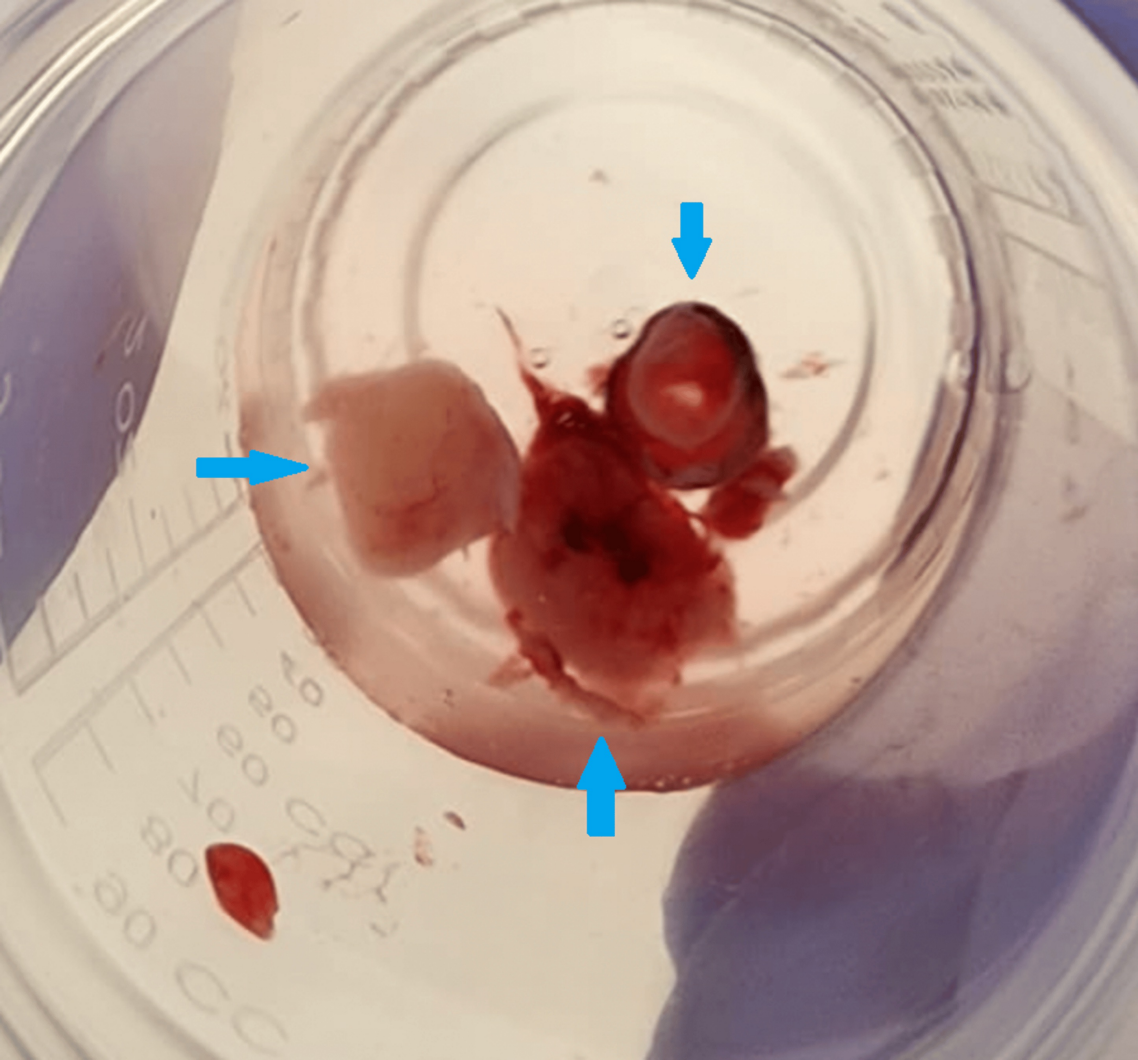
Gross specimens (blue arrows) of the mass after excision.

There were no issues with recovery from anesthesia, and the patient was discharged home on room air. Subsequent pathological analysis revealed multiple tan-white tissue fragments measuring 2.5 x 2.5 x 0.3cm in aggregate (Figure 6). Tumor cells stained positive for smooth muscle actin (SMA) and desmin, confirming the diagnosis of a benign leiomyoma.

## Discussion

Tracheal leiomyomas are extremely rare, with the first case described in the medical literature in 1955 [7]. Since then, case reports make up the bulk of the literature however a few single institution case series have been published - 16 cases published by Park et al. [1], 16 cases by Jhun et al. [8], 13 cases by Kim et al. [9], and 10 cases by Kwon et al. [10].

Symptoms vary depending on the size and location of the tumor. These tumors are typically endobronchial (estimated to be 45%), with the remaining occurring in the trachea or lung parenchyma [11]. While these tumors can be seen at any age, most patients present between the ages of 20 and 50 [12]. Initial diagnosis is often delayed due to a lack of symptoms or being misdiagnosed as asthma, with specific symptoms including recurrent wheezing, coughing, stridor, and shortness of breath [10]. Our patient was fortunate enough to be diagnosed early and only complained of asthma-like symptoms for a few months prior to her tracheal mass being incidentally discovered on imaging.

Radiological findings are non-specific and similar to other tracheal masses. Chest computed tomography (CT) typically shows a soft-tissue mass arising from the tracheal wall. CT can help define severity by estimating the degree of tracheal occlusion [13]. However, definitive diagnosis can only be made on histopathological examination.

Benign leiomyomas have an excellent outcome; however, they are rare and are far outnumbered by their malignant counterpart. These lesions need to be sampled and evaluated for cellular atypica, mitotic activity, and necrosis to differentiate between benign and malignant tumors [14]. Histological findings of leiomyomas include spindle-shaped smooth muscle cells within eosinophilic cytoplasm. Immunohistochemical markers can assist with the diagnosis and reflect smooth muscle markers, including smooth muscle actin (SMA), desmin, and h-caldesmon. Other markers, including HMB-45 (suggesting angiomyolipoma), S-100 (suggesting neural origin), cytokeratins (suggesting epithelial origin), and CD117 and CD34 (suggesting gastrointestinal stromal origin), are usually negative [15].

Both imaging and pathology can be useful in differentiating other types of smooth muscle airway tumors - most importantly, pulmonary leiomyosarcomas and benign metastasizing leiomyoma. Pulmonary leiomyosarcomas are malignant with histological features that include nuclear atypia, necrosis, vascular invasion, and high mitotic rate [16]. Benign metastasizing leiomyomas originate from the uterus and often present with multiple, bilateral lesions on imaging [17]. In addition, they are distinct in that they stain positive for estrogen and progesterone receptors on immunohistochemistry and may respond to hormonal treatment rather than surgical resection [18]. Our patient’s low-grade histology, along with positive immunohistochemical stains of SMA and desmin, and lack of multifocal nodules, were consistent with pulmonary benign leiomyoma.

Before diagnosis, it was noted that our patient had greater than 95% of luminal occlusion. While she did not have more severe symptoms of stridor or airway compromise, a careful airway approach was deemed necessary. There is a significant risk of airway compromise during diagnosis or treatment in patients with tracheal masses, causing complete airway occlusion (CAO), defined as a 50% or greater occlusion of the trachea, mainstem bronchi, bronchus intermedius, or lobar bronchi [4]. Manipulating the airway and tracheal space, while maintaining adequate ventilation, is challenging, and there exists a significant possibility of complete airway occlusion from excision or bleeding. Multiple considerations must be taken into account to minimize risk. Current American College of Chest Physicians (CHEST) guidelines recommend general anesthesia over moderate sedation and the use of rigid bronchoscopy either with jet or assisted ventilation for therapeutic interventions. The choice of awake vs asleep intubation depends on the severity and location of obstruction [4]. Laryngeal mask airway (LMA) offers another viable option for both surgical and bronchoscopic management and may be preferred in spontaneously ventilating patients. A recent single-center retrospective study showed 99.1% successful LMA ventilation in high-grade laryngotracheal stenosis undergoing surgical intervention [19]. While no study has looked at LMA specifically for CAO, a recent systematic review of LMA use during bronchoscopic intervention of airway stenosis showed low rates of intraoperative hypoxia (3.6%) and technique failure (0%), defined as requiring supplemental ventilation strategies [20]. Knowing the size and shape of our patient’s tumor and the impossibility of tracheal intubation, we elected for LMA placement while maintaining spontaneous ventilation. Additionally, rigid bronchoscopy was deferred, and a therapeutic bronchoscope (6.0mm outer channel, 2.7mm working channel) was utilized.

Given our patient’s CT findings, the procedure was deemed high risk, and an ECMO plan was developed by our intensivists, cardiac surgeons, and anesthesiologists. Criteria for ECMO initiation vary across centers, and no standardized framework currently exists for patients with central airway occlusion. While multidisciplinary discussion remains essential, we propose a structured framework to help guide clinical decision-making (Table 1). Our institution’s ECMO program has rapidly expanded since 2019, when deployment of intensivist-led cannulation was initiated [21]. Since then, our center has become an Extracorporeal Life Support Organization (ELSO) Gold Level Center of Excellence, averaging >40 cannulations per year. The ELSO Gold Level Center of Excellence is a prestigious international distinction awarded by ELSO to hospitals that provide exceptional ECMO care. Despite our proficiency, ECMO has its own risks and is associated with frequent complications, the most common being bleeding, occurring in 24-28% patients, and thrombosis, occurring in 35-58% of patients [22]. With careful consideration, ECMO backup was deemed the most optimal choice.

**Table 1:** Proposed Framework for ECMO in Central Airway Occlusion * Anticipated difficult airway factors: prior difficult airway, obesity, short neck, limited neck extension, Mallampati score > III. A proposed framework for extracorporeal membranous oxygenation (ECMO) utilization in central airway occlusion, derived from our institutional experience, established clinical principles, and prior literature emphasizing the importance of airway risk stratification and multidisciplinary pre-operative planning [4,5]. Table Credits: Liou J, Habib G, Desai R, Abouzgheib W.

Proposed Framework for ECMO in Central Airway Occlusion	
Risk Stratification	• Central airway occlusion > 75%
	• Anticipated difficult airway*
	• Acute or impending respiratory failure
	• ASA (American Society of Anesthesiologists) score > 3
Pre-Operative Multidisciplinary Planning	• ECMO team on standby in close vicinity
	• Ultrasound assessment of cannulation sites
	• Pre-specification of cannula type and sizing
	• Transesophageal echocardiography positioning and availability
	• ECMO circuit preparation (pump, oxygenator, heater, tubing)
Criteria for ECMO Deployment	• SpO₂ < 85% for > 2 minutes
	• Complete airway obstruction
	• Inability to bypass obstruction with small endotracheal tube
	• Hemodynamic instability

## Conclusions

Tracheal leiomyomas are rare benign tumors that can be diagnosed with bronchoscopic or surgical measures. Current guidelines recommend a bronchoscopic approach for resection of central airway tumors, although surgical resection can be considered due to the risk of incomplete resection or recurrence. Due to their slow growth, most patients are asymptomatic until the tumor is large and poses a significant risk of airway obstruction. A careful multidisciplinary approach must be considered to minimize potential complications. ECMO carries its own risks and complications; however, can be utilized as a rescue therapy in high proficiency centers.
